# Adherence to Combined Healthy Movement Behavior Guidelines among Adolescents: Effects on Cardiometabolic Health Markers

**DOI:** 10.3390/ijerph19148798

**Published:** 2022-07-20

**Authors:** Dartagnan Pinto Guedes, Marizete Arenhart Zuppa

**Affiliations:** 1 Health Sciences Center, Northern Parana University, Londrina 86041-100, Brazil; 2 Physical Education Center, Western Santa Catarina University, São Miguel do Oeste 89600-000, Brazil; marizetearenhartzuppa@hotmail.com

**Keywords:** adolescent behavior, health habits, health-promoting behaviors, youths

## Abstract

Background: The combined movement behavior guidelines for adolescents recommend ≥60 min of moderate to vigorous physical activity, ≤2 h of screen time, and 8–10 h of sleep. Considering that the information available on this topic in the young Latin American population is rare, this study aimed to identify the proportion of a sample of Brazilian adolescents meeting individual guidelines as well as the combination of the three healthy movement behavior guidelines. In addition, another objective of the study was to examine the effects of compliance with these guidelines on cardiometabolic health markers. Methods: This is a cross-sectional school-based study, with the participation of 306 adolescents aged 14 to 18 years. A questionnaire with structured questions was applied to collect data on physical activity, screen time, and sleep duration. Cardiometabolic health was assessed by the calculation of a continuous risk score, including twelve markers related to body fat, blood pressure, plasma lipids and lipoproteins, glycemia, and insulin. Results: Only 4.8% (4.3–5.4) of the adolescents met the three healthy movement behavior guidelines, while 9.3% (8.4–10.4) of the sample did not meet any of the guidelines. No significant difference between sexes was found in the simultaneous compliance of the three movement guidelines. Adolescents who did not meet any of the movement guidelines were twice as likely to have higher cardiometabolic risk (OR = 2.05 (1.41–3.17)) than their peers who met all three guidelines. Conclusions: Considering the high proportion of adolescents who did not meet the movement behavior guidelines and the negative effects on cardiometabolic health, it is suggested that future policies and interventions should consider an integrated and holistic approach aimed at simultaneous actions of maximizing physical activity, minimizing screen time, and ensuring sufficient sleep duration.

## 1. Introduction

Physical activity, sedentary behavior, and sleep duration are lifestyle behaviors that, combined, form the movement profile in a 24 h daily cycle [1]. Independent of each other, the importance of the three behaviors to the physical, mental, and social health of youths is well documented in the literature [2,3,4]. In fact, these findings have supported international organizations to propose guidelines with recommendations for the quantity and nature of movement behaviors that can enhance youths’ health benefits [5,6,7].

An important systematic review identified worldwide the proposition of 50 guidelines for the young population specifically directed at physical activity, 22 at sedentary behavior, and 3 at sleep duration [8]. In this case, there seems to be consensus among the various guidelines proposed, suggesting that adolescents should engage in 60 min of physical activity of moderate and vigorous daily [5], avoid spending no more than two hours of recreational screen time [6], and accumulate 8–10 h of sleep per night [7].

Although isolated movement behavior guidelines have been widely used to identify possible effects on youths’ health, the integrative nature of the movement profile paradigm in the 24 h daily cycle suggests that it simultaneously complies with the guidelines around physical activity, sedentary behavior, and sleep duration, and can lead to more effective benefits [1]. Supporting this assumption, emerging evidence has shown that meeting combined movement behavior guidelines is closely associated with lower rates of overweight [9], higher cardiorespiratory fitness [10], and more favorable indicators of mental health [11] in childhood and adolescence.

Despite the growing concern and awareness of their health benefits, adolescents in different regions of the world have shown low adherence to healthy movement behavior guidelines [12,13,14,15,16]. In addition, some subgroups of the young population are at a greater risk of not meeting the guidelines’ recommendations. For example, usually, a greater proportion of boys engage in more physical activity [17] and sleep long enough [18,19] compared with girls. Age is inversely associated with physical activity [20] and sleep duration [21], while older adolescents spend more recreational screen time than their younger peers [22]. A lower economic status is associated with less physical activity [20], recreational screen time [22], and sleep duration [23]. Family structure (living with both parents or only with one parent) has also been shown to be associated with recreational screen time [24]. A lower quantity of girls and older adolescents meet the recommendations of the three movement behavior guidelines simultaneously [17].

In this context, less is known about adherence to the combined movement behavior guidelines among Latin American adolescents. As far as the authors know, the existing studies have been conducted predominantly in developed countries [9,11,13,17] and may not reflect the behavior of young people from developing countries. Specifically in the young Brazilian population enrolled in public high schools, it was identified that the highest proportion met the recommendation of sleep duration (41%), followed by recreational screen time (28.6%) and physical activity (25.1%), while adherence to all three recommendations was 3.1% [25]. It is likely that the low number of adolescents adhering to the 24 h movement guidelines results from the lack of health education programs to promote the adoption of healthy behaviors among adolescents across many schools in Brazil.

Furthermore, given the limited amount of studies on the effects of adherence to the movement guidelines, both individual and combined, on cardiometabolic health markers in the young population [10], the available findings are still inconclusive, and further studies on this topic are needed. Therefore, this observational cross-sectional study has two aims: (a) to identify the proportion of a sample of Brazilian adolescents meeting individual and combined healthy movement behavior guidelines, including physical activity, sedentary behavior, and sleep duration; and (b) to examine the effects of compliance with these guidelines on cardiometabolic health markers.

## 2. Materials and Methods

### 2.1. Study Design and Participants

An observational cross-sectional study was developed, following the STROBE (STrengthening the Reporting of OBservational studies in Epidemiology) guidelines [26]. Data analyzed were derived from the *Health Promoting School Project*, a cohort school study designed and implemented by the Federal Institute of Santa Catarina, São Miguel do Oeste Campus. The students’ participation in the project was by choice and parents’ or guardians’ authorization was obtained. Thus, out of the 418 participants initially registered in the project baseline, data were considered from 306 adolescents aged between 14 and 18 years (179 girls and 127 boys) who presented complete data equivalent to the variables of interest for the present study. The participants who self-reported health problems, tobacco, alcohol, or other drug use, or who were undergoing some type of specific diet that could induce changes in study variables, were excluded from the analysis. The intervention protocols were approved by the Research Ethics Committee of Western Santa Catarina University (Platform Brazil no. 3.412.665/2019) and the rights of all participants were safeguarded by the free and informed consent form signed by the student and their guardian. Data were collected between August and November 2019 by a team of researchers who knew the instrument and were trained in its procedures.

### 2.2. Movement Behaviors

Data equivalent to the movement behaviors were obtained through a questionnaire specifically constructed for this purpose, addressing items on physical activity, sedentary behavior, and sleep duration. The questionnaire was answered in a single session, individually by each of the participants, and at the own place and time of the class. The participants of the study received the questionnaire with instructions and recommendations for self-completion, and no time limit was established for completion. Any doubts expressed by the respondents were promptly clarified by the researcher who followed the data collection. The reliability of the questionnaire was analyzed by reapplying it to 10% of the subjects seven days later. All of the items demonstrated *a Cohen* concordance index ≥0.80.

The physical activity was identified by the formulation of the following question: “*In the last seven days, how often have you have performed moderate to vigorous physical activity for at least 60 min (consider any type of physical activity that has increased your heart and respiratory rate, such as walking quickly, running, pedaling, swimming, or other similar activities; and the total time, that is, it is not necessary that it has been 60 min followed, can add up the moments of the day you performed some kind of physical activity)?*” The answer options for the question were from “none” to “7 days”.

Sedentary behavior was treated by exposure to leisure screen time through the following question: “*In a typical or usual week, how many hours do you watch TV and/or use computer, tablet, smartphone for any activity that is not related to any kind of assignment or homework?*” A predefined time scale was provided for the response, in which the respondents indicated their option between six categories, ranging from “*none*” to “≥5 *h/day*”. The question considered separately the use of screen devices on weekdays and weekends (Saturday and Sunday). A weighted average involving the weekday and weekend data was used to identify the screen time per day. 

Data equivalent to sleep duration were also gathered considering weekdays and weekends, with reference to a typical or usual week, by means of the following questions: “*On weekdays and weekends (Saturday and Sunday): (a) at what time do you usually sleep? (b) and at what time do you wake up?*” In possession of the reports presented by the participants, sleep time was calculated on weekdays and weekends. A weighted average involving the weekday and weekend data was used to identify the duration of sleep per night. 

International public health guidelines on movement behaviors for adolescents recommend ≥60 min/day of moderate to vigorous physical activity [5], ≤2 h/day of leisure screen time [6], and 8–10 h/night of sleep [7]. Therefore, in the sequence, for data analysis, the answers to the questions presented by the participants were dichotomized into two strata in each movement behavior: “*meets the guidelines*” and “*does not meet the guidelines*”.

### 2.3. Cardiometabolic Health

The adolescents’ cardiometabolic health was measured using a continuous score including the following variables: systolic (SBP) and diastolic (DBP) blood pressure, body mass index (BMI), waist circumference (WC), serum triglycerides (TG), total cholesterol (TC), high-density lipoprotein cholesterol (HDL-C), low-density lipoprotein cholesterol (LDL-C), the ratio of TC to HDL-C (TC/HDL-C), fasting glucose (GLU), and plasma insulin (INS). The last two variables were used to define insulin resistance by the homeostasis model (HOMA) using the standard formula [27]:

HOMA = ((insulin (μIU/mL) × glucose (mg/dL))/405

The continuous cardiometabolic health score was calculated by aggregating the residue standardization (z scores) of each variable according to sex and age, through the following formula: ([continuous variable value − cutoff point]/standard deviation). The cutoff points and standard deviations used were based on the study by Stavnsbo and collaborators [28]. Considering that HDL-C is inversely related to cardiometabolic risk, its value was multiplied by −1. A lower continuous score is indicative of a healthier cardiometabolic profile.

SBP and DBP were measured by the auscultatory method using a mercury sphygmomanometer. With the adolescent sitting, after a minimum period of 5 min of rest, blood pressure was measured in the left arm. Two measurements were taken, considering the mean value of both measurements for calculation purposes. 

BMI was calculated as body weight (kg) divided by height squared (m^2^). Body weight was recorded to the nearest 0.1 kg, using a portable electronic scale (Type SECA 861), and height to the nearest 0.1 cm, using a portable stadiometer (Type SECA 285). Light indoor clothing could be worn, excluding shoes, long trousers, and sweaters. WC was measured at the coincident point of the midway between the lower rib margin and the iliac crest, using an inextensible anthropometric tape with a resolution of 1 mm (Type SECA 201).

For biochemical analysis, serum samples and commercial kits (DiaSys Diagnostic Systems, Germany), performed on the Miura 200 automated device (I.S.E., Rome, Italy), were used. Plasmatic lipid and blood glucose measurements, GLU, and INS were performed by collecting 10 mL of venous blood samples at the elbow crease after a 10–12 h fasting period between 07:00 and 08:00. The serum was immediately separated by centrifugation; TG, TC, and HDL-C were measured by the enzymatic method; and LDL-C was measured using the *Friedewald* formula [29]. The TC/HDL-C ratio was also calculated. The GLU rate was analyzed using the hexokinase method and serum INS concentration was measured using an enzyme-linked immunosorbent assay.

### 2.4. Covariates

Sociodemographic data and food intake were included as covariates because of the relationship with movement behaviors and cardiometabolic variables [10,25]. With respect to demographic data, in addition to sex and age, information on year of study, parents’ schooling level, and family economic class was included, based on housing conditions, household utensils, cars, and number of domestic employees, according to Brazil classification criteria recommended by the Brazilian Association of Research Companies [30].

Regarding food intake, the participants stated how often they consume fruit/vegetables and sweetened products/soft drinks through the following questions: (a) “*In the last seven days, how often have you eaten fruits and/or vegetables?*” and (b) “*In the last seven days, how often have you*”drunk a bottle, can, or cup of soda and/or eaten cake, pie, cookies, sweets, or similar? The answer options for both questions were from “*none*” to “*seven days*”.

### 2.5. Data Analysis

Data analysis was conducted using the *IBM^®^ SPSS^®^ Statistics for Windows Package*, version 27 (IBM Corporate, Armonk, NY, USA). Regarding the movement behaviors (physical activity, sedentary behavior, and sleep duration), point proportions and respective confidence intervals (IC 95%) were identified, stratified according to sex. Statistical differences among strata under investigation were analyzed using a non-parametric chi-square test (χ^2^) for linear tendency. With respect to cardiometabolic health markers, the frequency distribution was initially analyzed using the *Kolmogorov–Smirnov* test. In this case, data on the TC/HDL-C ratio and TG concentration were transformed by natural logarithm because of its asymmetric distribution. Other variables showed a normal frequency distribution. Mean and standard deviation values were calculated and *Student’s t*-test for independent samples was used to establish comparisons between both sexes. Statistical significance was pre-established at *p* < 0.050.

Comparisons between continuous cardiometabolic health scores of the adolescents who met the movement behaviors guidelines were performed using analysis of covariance (ANCOVA) with adjustments for sociodemographic data and food intake. The chance of adolescents presenting a higher risk score for cardiometabolic health associated with movement behaviors (physical activity, sedentary behavior, and sleep duration) was identified by means of *odds ratio* values accompanied by 95% confidence intervals, calculated by binary logistic regression with adjustments for sociodemographic data and food intake. For this purpose, the sample was dichotomized based on the terciles’ distribution according to specific cutoff points by sex. The group with the highest risk for cardiometabolic health included adolescents with scores above the third tercile.

## 3. Results

The study participants had an average age equivalent to 16.34 ± 1.21 years. The demographic data of the selected sample are shown in Table 1.

Statistical information on cardiometabolic health markers and the proportions of adolescents who met the movement behaviors guidelines are available in Table 2. When comparing the mean values for most of the cardiometabolic health markers analyzed, no statistically significant differences were found between sexes. However, mean values equivalent to SBP were observed to be significantly higher in boys (121.87 vs. 114.9; *p* < 0.001) and plasma lipoproteins levels were statistically higher in girls (TC: 4.52 vs. 4.24; *p* = 0.021; HDL-C: 1.6 vs. 1.44; *p* < 0.001; LDL-C: 2.48 vs. 2.35; *p* = 0.039). The continuous cardiometabolic health scores were shown to be similar between the sexes.

Regarding the proportion of adolescents who adhered to guidelines of movement behaviors, overall, 9.3% [8.4–10.4] of the sample did not meet any of the guidelines, 65.5% [60.1–71.8] met only one guideline, 20.4% [18.3–22.8] met two guidelines, and 4.8% [4.3–5.4] of the sample met all three guidelines. A higher proportion of boys reported meeting physical activity guidelines alone (28.3% vs. 15.6%; *p* < 0.001) and the combination of physical activity and sleep duration (18.5% vs. 10.1%; *p* < 0.001). Girls reported significantly greater compliance with the recreational screen time guidelines alone (24% vs. 19.7%; *p* = 0.036) and the combination of leisure screen time and sleep duration (13.5% vs. 10.1; *p* = 0.019). Furthermore, the proportion of girls who met only a single guideline was higher (68.8% vs. 61.1%; *p* = 0.041), while the number of boys who met two guidelines together was higher (24.1% vs. 17.6%; *p* = 0.012).

Table 3 shows comparisons between the continuous cardiometabolic health scores of the adolescents who met and who did not meet the guidelines of healthy movement behaviors. The ANCOVA results with adjustments for sex and age revealed that individual compliance with each of the healthy movement behaviors did not result in significant differences in the continuous cardiometabolic health scores. However, adolescents who jointly met the physical activity and leisure screen time guidelines, the physical activity and sleep duration guidelines, and the three healthy movement behavior guidelines presented positive and significant effects on continuous cardiometabolic health scores.

Binary logistic regression models that reflect the chance of adolescents having a higher continuous risk score for cardiometabolic health associated with the guidelines of healthy movement behaviors are presented in Table 4. Each possible combination of complying with the guidelines of healthy movement behaviors was considered as an independent variable of interest, while meeting the three guidelines of healthy movement behaviors together was used as a reference for comparison in each model. The odds ratio values adjusted for sex and age suggest that adolescents who meet only the leisure screen time guideline (OR = 1.54 [1.06–2.36]; *p* = 0.038), the sleep duration guideline (OR = 1.68 [1.12–2.64]; *p* = 0.023), the guidelines equivalent to leisure screen time and sleep duration simultaneously (OR = 1.47 [1.02–2.25]; *p* = 0.046), or those who did not meet any of the guidelines (OR = 2.05 [1.41–3.177]; *p* < 0.001) were shown to be significantly more exposed to cardiometabolic health risks than their peers who reported meeting the three guidelines for healthy movement behaviors.

## 4. Discussion

The study produced a disturbing result, considering that a small proportion of adolescents reported adhere to the movement behavior guidelines and, therefore, could be enjoying their health benefits [9,10,11,12,13,14,15,16]. Less than 4.8% of the adolescents met the three movement behavior guidelines together, while twice the proportion of adolescents (9.3%) did not meet any of the three guidelines. Even considering that differences in adherence to the movement behavior guidelines among the studies should be interpreted with caution because of possible discrepancies in the approaches used to gather and analyze the data, the findings of the present study are similar to those presented by Canadian adolescents [31], slightly higher than that found in European [32] and Asian adolescents [33], but lower than that found in American adolescents [1]. Thus, these results highlight the importance of addressing the movement behaviors from an integrative and holistic approach, in an attempt to increase physical activity and sleep duration, while attempting to reduce recreational screen time [34].

Specifically, regarding the guidelines for physical activity, one in each group of five adolescents met the proposed recommendation (21.2%), with boys reporting significantly greater adherence than girls. Corroborating these results, an epidemiological study involving 1.6 million adolescents from different regions of the world revealed that 77.6% of boys and 84.7% of girls did not meet physical activity guidelines; in addition, the differences between sexes in meeting physical activity guidelines have increased in the last two decades in all regions of the world [35]. Therefore, promoting physical activity, with a greater emphasis on girls, is one of the main challenges faced today in the global public health of the young population. Considering that trends of meeting physical activity recommendations are decreasing worldwide, innovative policies and action strategies are necessary to meet this demand.

The screen-time-based sedentary behavior guideline was reached by 22.2% of the adolescents. Consistent with previous findings [35], in the present study, girls were more likely to comply with this guideline than boys. The proportion of youths meeting leisure screen time guidelines varies substantially among studies available in the literature [36]. Specifically, a range between 25.7% and 40.1% was found in European adolescents aged 14 to 18 years [32,37]. In principle, the differences among the studies can be explained by the type of measurement instrument used for data collection. While some studies sought to identify only TV exposure time, others identified the time spent using a wide range of screen devices. Surveys carried out over the last 20 years have shown the incentives received by the young population for the use of screen devices such as TVs, computers, tablets, and mainly *smartphones* [36], which justifies the difficulty of many adolescents meeting the leisure screen time guideline. Moreover, data available from previous studies point to differences between sexes regarding the use of screen devices. Generally, boys tend to spend more time on computers and tablets, while girls spend more time using *smartphones* [38].

Nearly one-third of the adolescents selected in the study met the guidelines for sleep duration (34.9%). Unlike the guidelines for physical activity and sedentary behavior based on recreational screen time, no differences were found between sexes in compliance with the sleep duration guideline. Other studies have shown that between 25.7% and 40.1% of adolescents from more developed countries meet the recommendations for sleep duration [32,37]. However, studies conducted in Spain identified that around 80–90% of a sample of adolescents met the sleep duration guideline [39]. Sleep duration is critical to the well-being of adolescents. Previous studies have shown that a lack of sleep during adolescence can result in impairment of mental health, through the appearance of symptoms of depression and anxiety, and inappropriate decisions about health care, including hyperphagia; physical inactivity; and the use of drugs such as caffeine, nicotine, or other stimulants [40].

On the other hand, the conceptualization of the three movement behaviors as co-dependent and integrated entities represents an important advance in the paradigm of health promotion and education in the young population. The findings of the current study contribute to strengthening this concept by pointing out that adolescents who separately met the physical activity, sedentary behavior, or sleep duration guidelines did not show continuous cardiometabolic health scores more favorable than their peers who did not meet the respective guidelines. However, adolescents who met the physical activity guideline in combination with the guidelines of sedentary behavior and/or sleep duration demonstrated lower chances of presenting a higher risk for cardiometabolic health, identifying compliance with the physical activity guideline as essential to the youths’ health. In addition, the adolescents who did not meet any of the movement behaviors guidelines presented a two-fold higher probability of cardiometabolic risk in comparison with adolescents who met all three guidelines together.

As far as it is known, only two other previous studies have specifically examined the association between compliance with the guidelines of movement behaviors and cardiometabolic markers in the young population [10,41]. In this case, the findings of the present study are corroborated by the results of the study performed with young Canadians, in the sense that the joint adherence to two or more guidelines of movement behaviors was associated with healthier individual scores of cardiometabolic markers [10]. However, they differ from the results found in the study performed with young Americans, in which a lower effect of simultaneous compliance with the three guidelines of movement behaviors on selected cardiometabolic markers was identified [41]. Several factors may contribute to the divergences in results found among the studies, including the age range of the young population considered, the methodology used to identify the movement behaviors, the different sets of cardiometabolic markers selected, and the time of data collection.

In addition to these studies that specifically sought to examine the guidelines of movement behaviors, other studies reported the effect of different combinations of lifestyle behaviors on cardiometabolic markers in young people. For example, using data from approximately twenty-one thousand youths aged 4 to 18 years, it was identified that higher physical activity is associated with lower levels of SBP and TG and higher levels of HDL-C in sedentary time terciles; however, no association was identified between sedentary time and cardiometabolic markers after adjustment for physical activity [42]. In a longitudinal study, the impact of changes in multiple lifestyle behaviors on cardiometabolic markers in young Danish was examined. The results showed that the participants grouped in the most favorable tercile of changes related to physical activity and sleep duration presented a significant reduction in a score composed of metabolic syndrome in comparison with their peers grouped in opposite terciles [43].

Given the low adherence of the study participants to the combined guidelines of physical activity, screen time-based sedentary behavior, and sleep duration, and their effects on cardiometabolic health markers, special attention should be given to programs to encourage movement behaviors. In this sense, the school may be an appropriate environment for the promotion of adolescents’ health behaviors. It is noteworthy that the literature presents strong evidence that chronic non-transmissible diseases, manifested in adulthood, result from complex interactions between a variety of cardiometabolic health markers that may originate in childhood and adolescence [44]. Therefore, young people who are eventually more exposed to cardiometabolic health risks, with advancing age, tend to be more predisposed to the onset of cardiovascular disease and diabetes mellitus. Thus, early detection of the presence of cardiometabolic health risk markers in the young population is defined as an important primary care strategy that can effectively contribute to the prevention of chronic outcomes in adulthood and reduce public health expenditures.

In this case, ideally, the design and implementation of strategies and programs to promote health behaviors in the school context should involve the participation of both teachers and school managers, as well as students’ families [45]. For example, restricting the use of media and the presence of screen equipment in the bedroom, promoting participation in active play outside the school environment and active travel, making physical education classes more physically active, and encouraging active leisure in school breaks may be some strategies to be used to reallocate sedentary time to active behaviors and adequate sleep duration.

The study presents some strengths and limitations that should be mentioned. The use of a continuous score grouping twelve cardiometabolic health markers, as opposed to the presence/absence dichotomous of individual risk factors, is an important point to be highlighted, considering that it allows to measure the effect of combined movement behaviors on cardiometabolic health in healthier young people, rather than simply identifying these effects in youths who reach the threshold of pathological diagnosis. Another strong point is the fact that the possible seasonal interferences in the data of adolescents were minimized considering that their collection was carried out in a short period of time (three months) and in the same season of the year (spring), which, together with a minimum refusal rate to participate in the study, ensures greater reliability of the findings. 

Regarding the probable limitations, the collection of information about the movement behaviors was carried out by means of self-reported questionnaires, which are limited by memory and may present bias due to the possible use of more desirable statements. Moreover, only the use of screen devices was included in sedentary behavior. However, in comparison with other sedentary behavior modalities, screen time tends to present a greater variation among young people and more effective voluntary control. In addition, the cross-sectional nature of the data may limit the inferences of cause and effect of the movement behaviors on cardiometabolic health. The subject of future study is to examine the temporal stability in meeting the guidelines of movement behaviors and its effects on the adolescents’ cardiometabolic health through the use of a longitudinal study design. Residual confusion caused by unidentified and unmeasured factors may in some way enhance the inaccuracies of the findings. The low proportions of adherence to the guidelines of the combined movement behaviors require careful interpretation of *p*-values, because the tests performed are asymptotic and only approximate. Finally, the sample selected is representative of the population of high school adolescents of the western region of Santa Catarina State, Brazil. For this reason, although a careful delimitation, definition, and selection of the sample was used, the results should not be generalized to populations of adolescent from different socio-cultural environments and economic development.

## 5. Conclusions

In conclusion, the study revealed that only a small proportion of adolescents met the combined guidelines of movement behaviors (physical activity ≥ 60 min/day, leisure screen time ≤ 2 h/day, and sleep duration equivalent to 8–10 h/night). Although boys reported greater compliance with the physical activity guideline and girls with the leisure screen time guideline, no significant difference was found between sexes in the joint adherence of the three movement behavior guidelines. The adolescents who met the physical activity guideline combined with the sedentary behavior and/or sleep duration guidelines demonstrated lower chances of presenting a higher risk for cardiometabolic health. Thus, it is suggested that future policies and interventions to promote adolescent health should consider an integrated and holistic approach aimed at simultaneous actions of maximizing the practice of physical activity, minimizing screen time, and ensuring sufficient sleep duration.

## Figures and Tables

**Table 1:** Demographic data of the selected sample in the study (*n* = 306).

	*n* (%)		*n* (%)
Sex		Parents’ schooling	
Girls	179 (58.5%)	≤5 years	62 (20.3%)
Boys	127 (41.5%)	6–11 years	129 (42.2%)
Age		≥12 years	115 (37.5%)
14–15 years	80 (26.1%)		
16–18 years	226 (73.9%)	Family economic class	
Year of Study		Low	37 (12.1%)
1st Year	117 (38.2%)	Intermediate	209 (68.3%)
2nd Year	106 (34.6%)	High	60 (19.6%)
3rd Year	83 (27.2%)		

**Table 2:** Descriptive characteristics of cardiometabolic health markers and movement behaviors according to the sex of the adolescents participating in the study.

	Both Sexes	Girls	Boys	*p*
Cardiometabolic health markers (Mean ± Standard deviation) ^1^				
Systolic blood pressure (mmHg)	117.79 ± 9.58	114.90 ± 9.28	121.87 ± 9.97	<0.001
Diastolic blood pressure (mmHg)	69.36 ± 8.44	69.55 ± 8.32	69.10 ± 8.62	ns
Body mass index (BMI)	23.13 ± 4.37	23.30 ± 4.44	22.89 ± 4.27	ns
Waist circumference (cm)	79.85 ± 11.07	78.19 ± 10.88	80.79 ± 11.34	ns
Triglycerides (mmol/L)	0.88 ± 0.41	0.87 ± 0.40	0.89 ± 0.43	ns
Total cholesterol (mmol/L)	4.40 ± 0.80	4.52 ± 0.81	4.24 ± 0.79	0.021
HDL-C (mmol/L)	1.53 ± 0.33	1.60 ± 0.34	1.44 ± 0.34	<0.001
LDL-C (mmol/L)	2.42 ± 0.70	2.48 ± 0.71	2.35 ± 0.68	0.039
Total cholesterol / HDL-C	3.09 ± 0.82	2.94 ± 0.80	3.31 ± 0.86	<0.001
Fasting glucose (mmol/L)	5.39 ± 0.43	5.31 ± 0.43	5.51 ± 0.44	ns
Plasma insulin (pmol/L)	64.51 ± 25.52	65.53 ± 26.41	63.07 ± 24.27	ns
HOMA-IR	2.47 ± 1.37	2.46 ± 1.32	2.48 ± 1.45	ns
Cardiometabolic health score	0.02 ± 2.09	0.03 ± 2.04	0.01 ± 2.17	ns
Proportion of the sample that met the guidelines for movement behaviors (% [CI 95%]) ^2^				
Did not meet any guidelines	9.3 [8.4–10.4]	9.2 [8.4–10.2]	9.5 [8.5–10.7]	ns
Met a single guideline	65.5 [60.1–71.8]	68.8 [63.1–75.3]	61.1 [56.0–67.3]	0.041
Physical activity	21.2 [19.1–23.7]	15.6 [14.4–17.2]	28.3 [25.1–31.9]	<0.001
Screen time	22.2 [20.0–24.9]	24.0 [21.6–26.9]	19.7 [17.7–21.8]	0.036
Sleep duration	34.9 [31.1–39.4]	36.2 [32.0–40.9]	33.0 [29.4–37.2]	ns
Met two guidelines	20.4 [18.3–22.8]	17.6 [15.9–19.7]	24.1 [21.7–27.0]	0.012
Physical activity + screen time	8.5 [7.7–9.4]	7.8 [7.1–8.8]	9.6 [8.6–10.8]	ns
Physical activity + sleep duration	13.6 [12.3–15.2]	10.1 [9.1–11.3]	18.5 [16.7–20.7]	<0.001
Screen time + sleep duration	12.1 [10.9–13.5]	13.5 [12.2–15.1]	10.1 [9.1–11.3]	0.019
Met all three guidelines	4.8 [4.3–5.4]	4.4 [4.0–5.1]	5.3 [4.8–6.0]	ns

^1^ Comparison between both sexes using *Student’s t* test. ^2^ Comparison between both sexes using *chi-square* test. ns = no significant.

**Table 3:** Comparisons between the continuous cardiometabolic health scores of adolescents who met and who did not meet the guidelines of movement behaviors.

	Guidelines of Movement Behaviors		
	Meet	Did Not Meet	*p* ^1^
Physical activity	−3.25 ± 3.05	3.50 ± 3.23	ns
Leisure screen time	−0.97 ± 2.16	1.46 ± 3.74	ns
Sleep duration	−1.64 ± 3.72	1.11 ± 4.07	ns
Physical activity + leisure screen time	−5.57 ± 4.85	6.62 ± 4.93	0.012
Physical activity + sleep duration	−4.13 ± 3.48	5.28 ± 4.61	0.026
Leisure screen time + sleep duration	−2.58 ± 3.26	3.74 ± 5.12	ns
All three behaviors	−9.52 ± 7.39	7.84 ± 6.57	<0.001

^1^ ANCOVA with adjustments for sociodemographic data and food intake. ns = no significant.

**Table 4:** The chance of adolescents presenting a higher continuous risk score for cardiometabolic health associated with the guidelines of healthy movement behaviors (physical activity, recreational screen time, and sleep duration).

	Odds Ratio (CI 95%) ^1^	*p*
Meets the three movement behavior guidelines	Reference	
Physical activity	1.32 [0.91–2.03]	ns
Leisure screen time	1.54 [1.06–2.36]	0.038
Sleep duration	1.68 [1.12–2.64]	0.023
Physical activity + leisure screen time	1.17 [0.79–1.80]	ns
Physical activity + sleep duration	1.22 [0.84–1.89]	ns
Leisure screen time + sleep duration	1.47 [1.02–2.25]	0.046
Does not meet any of the movement behavior guidelines	2.05 [1.41–3.17]	<0.001

^1^ Values adjusted for sociodemographic data and food intake. ns = no significant.

## Data Availability

The data used are available within the manuscript.
